# Secondary Prophylaxis of Hepatic encephalopathy in Liver cirrhosis: Rifixamine plus Probiotics vs. Rifixamine alone

**DOI:** 10.12669/pjms.42.3.13066

**Published:** 2026-03

**Authors:** Khurram Shahzad, Shahid Sarwar, Samina Saeed, Amina Umer

**Affiliations:** 1 Dr. Khurram Shahzad, FCPS (Medicine) Post Graduate Resident, Department of Medicine, Jinnah Hospital, Lahore, Pakistan; 2 Dr. Shahid Sarwar, FCPS (Medicine), FCPS (Gastroenterology), MCPS-HPE, FRCP(Edin) Professor of Medicine, Department of Medicine, Jinnah Hospital, Lahore, Pakistan; 3 Dr. Samina Saeed, FCPS (Medicine) Professor of Medicine, Department of Medicine, Jinnah Hospital, Lahore, Pakistan; 4 Dr. Amina Umer, FCPS (Medicine), FCPS (Endocrinology) Associate Professor of Medicine, Department of Medicine, Jinnah Hospital, Lahore, Pakistan

**Keywords:** Cirrhosis, Hepatic encephalopathy, Probiotics, Rifaximin, Secondary prophylaxis

## Abstract

**Objective:**

To determine the efficacy of adding probiotics with rifaximin for the secondary prevention of hepatic encephalopathy in patients with liver cirrhosis.

**Methodology:**

In this qausi experimental study, conducted in Department of Medicine, Jinnah Hospital Lahore from December 2024 to May 2025, 120 patients with liver cirrhosis and history of at least one episode of hepatic encephalopathy were enrolled. Participants were allocated into two groups: Group-A received rifaximin 550 mg twice daily along with Saccharomyces boulardii-based probiotics twice daily, while Group-B was treated with rifaximin alone. All patients were followed up for six months to monitor for recurrence of hepatic encephalopathy. Data analysis was conducted using SPSS 25 and p-value of less than 0.05 was considered statistically significant.

**Results:**

Among 120 included patients with a mean age of 55.71years (±10.18) and a male-to-female ratio of 1.2 (66/54), hepatic encephalopathy recurred in nine patients (15%) in Group-A compared to 23 patients (38.3%) in Group-B, (p=0.004). The baseline characteristics, including liver disease severity and comorbidities, were comparable between the groups. Among patients who experienced recurrence of hepatic encephalopathy there were no significant differences in age, duration of liver disease, INR, serum creatinine, albumin levels, or presence of ascites, highlighting the treatment regimen as the primary factor influencing outcomes.

**Conclusion:**

The addition of probiotics to rifaximin therapy markedly decreased the recurrence of hepatic encephalopathy compared to rifaximin alone, suggesting a more effective strategy for secondary prevention.

## INTRODUCTION

Hepatic encephalopathy (HE) is a neuropsychiatric disorder resulting from liver dysfunction and portosystemic shunting, and it commonly occurs in individuals with liver cirrhosis. Its clinical manifestations range from subtle cognitive impairment to deep coma, making it a major contributor to hospitalizations and reduced quality of life.^1^-^3^ Hepatic encephalopathy is the most common cause of altered sensorium in patients with liver cirrhosis and is an important cause of recurrent hospital admissions in patients with cirrhosis.^2^

Hepatic encephalopathy arises due to the liver’s inability to detoxify various circulating toxins, particularly ammonia. These toxins can cross the blood-brain barrier, where ammonia is converted into glutamate within astrocytes, leading to cerebral edema and altered mental status.^4^ The pathogenesis of hepatic encephalopathy is multifactorial, involving ammonia, inflammation, and endotoxemia as key contributors. Additionally, gut microbiota dysbiosis in cirrhotic patients has been linked to hyperammonemia, systemic inflammation, neuroinflammation, and endotoxemia.^5^

Rifaximin, a non-absorbable oral antibiotic, has demonstrated efficacy in lowering the risk of HE recurrence by targeting and reducing ammonia-producing gut bacteria.^6^,^7^ However, it does not sufficiently address systemic inflammation and gut microbiota imbalances, collectively termed intestinal dysbiosis, which are integral to the pathogenesis of HE.^8^,^9^ Probiotics, defined as live microorganisms that offer health benefits to the host, help re-establish microbial balance, enhance gut barrier function, and diminish endotoxemia and systemic inflammation. Consequently, their role in HE prevention is increasingly being recognized.^9^

Evidence from clinical trials suggests that probiotic supplementation can improve neurocognitive performance and lower ammonia levels in cirrhotic patients with HE.^10^,^11^ The therapeutic rationale for combining rifaximin with probiotics stems from their synergistic mechanisms: while rifaximin suppresses harmful bacterial overgrowth, probiotics foster the growth of beneficial microbes and support mucosal integrity. Despite this potential, comparative data on their combined use for the secondary prophylaxis of HE remains limited. This study aimed to determine whether the addition of probiotics to rifaximin therapy provides enhanced protection against recurrent HE compared with rifaximin alone.

## METHODOLOGY

This prospective, quasi experimental study was conducted at the Gastroenterology Unit of Jinnah Hospital Lahore between December 2024 and May 2025. Sample size was calculated using the WHO sample size calculator using power of 80% and level of significance 5%.We included patients with decompensated cirrhosis of the liver with age range 18 to 65 years, including both males and females who recovered from at least one episode of hepatic encephalopathy Grade-2 or more according to the West Haven criteria. Patients with diagnosed neuropsychiatric disease or using psychoactive drugs (alcohol, anti-epileptics or anti-psychotics, those having used antibiotics in the four weeks before inclusion in the study, patients with acute or chronic renal disease and metabolic encephalopathy without liver involvement and patients with diagnosed HCC were excluded from study.

### Ethical Approval:

The study protocol was approved by the Institutional Review Board (ERB153/4/26-10-23/S1 ERB; dated October 26, 2023), and informed consent was obtained from all participants prior to enrollment.

Participants were divided into two groups (n=60 each) using an alternate assignment method. The first eligible patient was assigned to Group-A, the next to Group-B, and this pattern continued sequentially throughout the enrollment period. Group-A was started on Rifaximin 550 mg twice a day and Saccharomyces Boullardi-based probiotics twice a day along with standard of care therapy including lactulose, dietary protein restriction 1.2 gm/day and identification and treatment of risk factors, while Group-B received Rifaximin 550 mg twice a day along with standard treatment. Each sachet of probiotic given to Group-A contained saccharomyces Boullardi 250 mg (5×10^9^ CFU).

Patients were followed up for six months, with monthly assessments conducted to detect the recurrence of hepatic encephalopathy. Patients were assessed based on the West Haven criteria by the same trained physician on each follow up visit to reduce the observer bias. Laboratory investigations included measurements of the international normalized ratio (INR), serum albumin, creatinine, and platelet count. Additionally, abdominal ultrasound was performed to evaluate the presence of ascites and portal vein thrombosis.

### Statistical analysis:

Data were analyzed using SPSS version 25. Quantitative variables were described as mean± standard deviation and qualitative variables were expressed as percentage. Primary outcome measure was relapse of hepatic encephalopathy or completion of six month follow up. The chi-square test was applied to analyze categorical variables, while independent t-tests were used for continuous variables. A p-value of less than 0.05 was considered statistically significant. Group-A and B were compared for recurrence of hepatic encephalopathy using chi square (X^2^).

### Informed consent:

Consent was obtained from all participants prior to enrollment.

## RESULTS

We included 120 patients with liver cirrhosis, admitted with hepatic encephalopathy and recovered in our study, with a mean age of 55.71(±10.18) years and a male-to-female ratio of 1.23 (66/54). The median duration of illness in the included patients was 45 months (Range 1-120 months). Liver cirrhosis was diagnosed due to variceal bleeding in 35 (29.2%) patients, hepatic encephalopathy in 26 (21.7%) patients, 17(14.2%) were diagnosed due to the development of ascites, and 42 (35%) were noted to have chronic liver disease during routine screening. At the time of inclusion in the study, 83 (69.2%) patients had a history of variceal bleeding, and 92 (76.6%) had been treated for ascites.

All patients had a history of at least one episode of hepatic encephalopathy, with a maximum hepatic encephalopathy grade of two in 58 (48.3%), grade 3 in 46(38.3%), and Grade-4 in 16(13.3%) patients. Diabetes Mellitus was present in 32(26.7%) patients, while 27(22.5%) were hypertensive. Portal vein thrombosis was present in 26(21.7%) patients without hepatocellular carcinoma. Majority of the included patients were in Child Turcotte Pugh (CTP) class C 61(50.8%), 55(45.8%) were in CTP B, while only 4 (3.3%) patients were in CTP class A at the time of inclusion in the study.

Patients were divided into Group-A, who received rifaximin along with probiotics for secondary prophylaxis of hepatic encephalopathy in addition to standard of care, and Group-B, who were prescribed rifaximin in addition to standard treatment. Both groups were compared for disease-related variables in Table-I with no significant differences.

**Table-I:** Comparison of patients included in two study groups

Variables	Group-A n-60	Group-B n-60	P value
Male/Female	33/27	33/27	1
H/O GI bleeding	41	42	0.84
Grade of previous HE 2/3/4	30/23/7	28/23/9	0.85
Past H/O Ascites	46	46	0.97
Diabetes Mellitus	15	17	0.68
Hypertension	12	15	0.51
Portal vein thrombosis	14	12	0.65
CTP Class A/B/C	2/27/31	2/28/30	0.99
Age in years (Mean±SD)	54.90(10.45	56.52(9.94)	0.38
Platelet count/ x 103/µL	154.26	153.93	0.98
INR	1.32(0.27)	1.50(1.31)	0.31
Creatinine mg/dl	1.2(0.59)	1.21 (0.60)	0.95

All patients were followed up for six months, and recurrent hepatic encephalopathy developed in 32 (26.6%) patients; nine (15%) patients in Group-A and 23 (38.3%) in Group-B developed HE during the follow-up period with relative risk (RR) of developing recurrent HE 0.28 (0.11-0.68) for those receiving both rifaximin and probiotic and the difference was significant (p = 0.004). (Table-II) We compared patients with recurrent HE in follow-up and those with no HE in six months, as shown in Table-III for variables related to the severity of underlying liver disease, and found no significant difference, highlighting the fact that significant differences in outcome are due to the intervention in the form of adding probiotics to rifaximin for secondary prophylaxis of hepatic encephalopathy in patients with cirrhosis.

**Table-II:** Risk Estimate for Recurrent PSE

	Value	95% Confidence Interval	
		Lower	Upper
Odds Ratio for Treatment given (Rifixamine and Probiotics (Group-A) / Rifixamine alone (Group-B))	0.284	0.118	0.684
For cohort Recurrent HE = Yes	0.391	0.198	0.774
For cohort Recurrent HE = No	1.378	1.100	1.728
N of Valid Cases	120		

**Table-III:** Comparison of patients who developed PSE with those who did not developed PSE during follow up.

Variable	PSE during follow up (n-32)	No PSE during follow up (n-88)	P-value
Age (Years)	57 (7.72)	55.24 (10.96)	0.4
Duration of illness (Months)	42	46	0.68
INR	1.25(0.20)	1.47(1.09)	0.3
Albumin (g/dl)	2.57 (0.63)	2.73 (0.73)	0.75
Creatinine (mg/dl)	1.14 (0.62)	1.24 (0.58)	0.39
H/O GI bleeding	24	59	0.4
Grade of HE before inclusion (2/3/4)	15/13/4	43/33/12	0.95
Ascites before inclusion in study (Mild/Mod/Severe)	3/18/3	27/37/4	0.09
Portal vein thrombosis on US	4	22	0.09

## DISCUSSION

Our study clearly demonstrates that the combined use of probiotics and rifaximin results in a significantly lower recurrence of hepatic encephalopathy than treatment with rifaximin alone. Specifically, patients receiving combination therapy exhibited Relative Risk of 0.28 as compared to patients with rifaximin alone, underscoring the added therapeutic value of addressing both ammonia accumulation and gut microbial imbalance in patients with cirrhosis.

These results align with previous clinical studies that reported notable improvements in both minimal hepatic encephalopathy (MHE) and overt hepatic encephalopathy episodes following probiotic administration.^1^,^10^ Additionally, improved cognitive function has consistently been observed in patients undergoing probiotic therapy.^11^ Further supporting evidence highlights the synergistic effect of combining rifaximin with probiotics, particularly in attenuating systemic inflammation and reinforcing the gut-liver-brain axis, which plays a crucial role in the pathophysiology of HE.^9^,^10^

Although rifaximin is effective in reducing the burden of ammonia-producing bacteria in the gut, it does not fully restore intestinal barrier integrity or microbiota diversity.^8^,^12^ In contrast, probiotics contribute to strengthening epithelial function, modulating immune responses through anti-inflammatory cytokines, and lowering endotoxemia, all of which are instrumental in preventing HE recurrence.^13^ Emerging strategies, such as fecal microbiota transplantation (FMT), have also shown promise in modulating the gut microbiome and improving HE outcomes.^14^

Multiple meta-analyses support the role of probiotics in mitigating the severity and recurrence of HE.^10^,^11^ Moreover, real-world data and systematic reviews suggest that the combined approach not only reduces hospital readmissions but also enhances neurocognitive recovery and quality of life in affected patients.^9^,^12^ To strengthen the generalizability of these findings, future research should involve multicenter, double-blind randomized trials, diverse probiotic strains with larger sample sizes and longer follow up.

### Limitations:

Despite these encouraging results, this study had certain limitations. It was conducted at a single center and utilized an open-label design, which may have introduced bias. Additionally, only one probiotic formulation was evaluated, and outcomes may differ depending on strain specificity, dosing regimens, or patient populations. Patients were followed for six months for efficacy of probiotics, longer follow up time can add further authenticity to conclusions. Multivariate analysis was not performed because of limited sample size and insufficient number of outcome events, but baseline characteristics were compare-able between two groups and results should be interpreted as suggestive rather than definitive.

## CONCLUSION

The adjunctive administration of probiotics alongside rifaximin may reduce the risk of hepatic encephalopathy recurrence in patients with cirrhosis. This combined approach is suggestive of a promising and potentially superior strategy for secondary prophylaxis. Incorporating probiotics into routine management may lead to better clinical outcomes, reduced recurrence rates, and lower burden on healthcare resources.

### Authors` contribution:

**KS:** Conception and design, data collection and manuscript writing.

**SS:** Conception, data analysis, manuscript review.

**SS, & AU**: Design of study, data collection, manuscript review.

All authors have approved the final version and are also accountable for the integrity of the study.
